# External quality assessment scheme for sperm DNA fragmentation: a pilot study in China

**DOI:** 10.1186/s12610-023-00211-0

**Published:** 2023-11-28

**Authors:** Yan Zheng, Ying-Bi Wu, Ye-Lin Jia, Li-Juan Ying, Ting-Ting Yang, Qing-Yuan Cheng, Jiao Qin, Chen Luo, Lin Yu, Fu-Ping Li

**Affiliations:** 1 Department of Andrology/Sichuan Human Sperm Bank, West China Second University Hospital, Sichuan University, Chengdu, 610041 P.R. China; 2 Key Laboratory of Birth Defects and Related Diseases of Women and Children (Sichuan University), Ministry of Education, Chengdu, 610041 P.R. China

**Keywords:** External quality control, Sperm DNA fragmentation, Sperm chromatin structure assay, Sperm chromatin dispersion, Contrôle externe de Qualité, Fragmentation de l’ADN des Spermatozoïdes, Test de Structure de la Chromatine des Spermatozoïdes, Test de Dispersion de la Chromatine des Spermatozoïdes

## Abstract

**Background:**

The aim of this article is to establish an external quality assessment (EQA) scheme for sperm Deoxyribonucleic acid (DNA) fragmentation (SDF) detection, and to assess the feasibility of the scheme. In addition, this article provides some case analysis of abnormal results in order to really help improve the performance of the laboratory.

**Results:**

In 2021 and 2022, 10 and 28 laboratories in China volunteered to participate in the EQA program respectively. Two samples were selected for EQA each year, a large spread of results was obtained for the four samples, and the highest values were 13.7, 4.2, 8.0 and 4.0 times the lowest respectively. The coefficients of variation (CVs) were very high for the four samples, at 46.6%, 30.1%, 26.7% and 30.3%, respectively. The CVs of the samples with high SDF values were lower than those of the samples with low SDF values. There was no significant difference between the results of sperm chromatin structure assay (SCSA) and sperm chromatin dispersion (SCD). For the 10 laboratories that participated in EQA in 2021 and 2022, the CVs of low SDF value samples and high SDF value samples decreased from 46.6% and 30.1% in 2021 to 32.5% and 22.7% in 2022, respectively.

**Conclusion:**

This is the first study to evaluate the EQA program on SDF, which involved a number of laboratories and was demonstrated to be feasible. It is recommended that all laboratories participate in the EQA of SDF to ensure the accuracy of the results.

**Supplementary Information:**

The online version contains supplementary material available at 10.1186/s12610-023-00211-0.

## Résumé

**Contextes:**

L’objectif de cet article est d’établir un système externe d’évaluation de la qualité (EEQ) pour la détection de la fragmentation de l’ADN des spermatozoïdes (SDF) et d’évaluer la faisabilité de ce système. En outre, cet article fournit une analyse de cas de résultats anormaux afin d’aider réellement à améliorer les performances du laboratoire.

**Résultats:**

En 2021 et 2022, respectivement 10 et 28 laboratoires en Chine se sont portés volontaires pour participer au programme EEQ. Deux échantillons ont été sélectionnés chaque année pour l’EEQ ; un large éventail de résultats a été obtenu pour les quatre échantillons, et les valeurs les plus élevées étaient respectivement de 13,7, 4,2, 8,0 et 4,0 fois les plus faibles. Les coefficients de variation (CV) étaient très élevés pour les quatre échantillons, soit respectivement 46,6 %, 30,1 %, 26,7 % et 30,3 %. Les CV des échantillons avec des valeurs de SDF élevées étaient inférieurs à ceux des échantillons avec de faibles valeurs de SDF. Il n’y avait pas de différence significative entre les résultats du test de structure de la chromatine des spermatozoïdes (SCSA) et ceux de la dispersion de la chromatine des spermatozoïdes (SCD). Pour les 10 laboratoires qui ont participé à l’EEQ en 2021 et 2022, les CV des échantillons à faible valeur de SDF et ceux des échantillons à valeur élevée de SDF ont diminué, passant respectivement de 46,6 % et 30,1 % en 2021 à 32,5 % et 22,7 % en 2022.

**Conclusions:**

Il s’agit de la première étude à évaluer le programme externe d’évaluation de la qualité (EEQ) de l’analyse de la SDF, qui a impliqué un certain nombre de laboratoires, et qui s’est avéré réalisable. Il est recommandé que tous les laboratoires participent à l’EEQ de la SDF afin d’en assurer l’exactitude des résultats.

## Introduction

More than 15% of couples worldwide are infertile, with male factors alone or in combination with female factors contributing to 50% of infertility [1]. Currently, the assessment of male infertility primarily relies on basic semen analysis, which includes evaluating the concentration, motility, and morphology of sperm. However, it has been observed that semen analysis does not accurately predict male fertility potential [2]. Surprisingly, around 15% of infertile patients have completely normal semen analysis results, indicating that routine semen analysis parameters are not sufficient to identify all potential fertility issues in men [3]. One important aspect that semen analysis cannot effectively measure is sperm Deoxyribonucleic acid (DNA) damage, which refers to any chemical changes in the DNA structure of sperm. Among these changes, sperm DNA fragmentation (SDF) is one of the most common disturbances, characterized by single or double strand breaks in the genetic material. Since 1999, there has been a significant increase in the number of studies on the relationship between SDF and male infertility [4]. This suggests that SDF may serve as a crucial complementary indicator of male infertility because it is partially correlated with semen quality. Furthermore, SDF has been proposed to be associated with infertility even in individuals with normal spermatozoa [5, 6]. In recent years, several meta-analyses have highlighted the adverse effects of SDF on various stages of reproduction, including embryo development, implantation, pregnancy, and the overall health of offspring in both natural and assisted reproduction [7–13].

Therefore, SDF has emerged as one of the most promising biomarkers in the field of andrology, and it is considered the most common infertility test besides semen analysis [14]. Several methods for detecting SDF are available, such as the terminal deoxynucleotidyl transferase dUTP nick end labeling (TUNEL) assay, comet assay, sperm chromatin structure assay (SCSA), and sperm chromatin dispersion (SCD). However, studies have shown that different methods yield different information and the results obtained from SCSA and SCD assays often conflict with each other [15, 16]. Therefore, it is crucial for laboratories to provide clinicians and patients with reliable and accurate SDF test results. For this reason, quality control is necessary for each laboratory test, with internal quality control (IQC) measuring the variability in laboratory results, and external quality assessment (EQA) being useful for detecting systematic variations and assessing accuracy. However, to the best of our knowledge, there had been no previous EQA on SDF organized and reported in China. To bridge this gap, we organized a national pilot EQA program for SDF in 2021–2022, involving 28 laboratories. The objective of this study is to establish an EQA scheme for SDF detection, and to evaluate the feasibility of the scheme. Additionally, this article provides case analyses of abnormal results to truly aid in improving the laboratory's performance in SDF testing.

## Materials and methods

### Scheme organization

In 2021 and 2022, 10 and 28 laboratories in China volunteered to participate in the EQA program. The pilot EQA program for SDF was organized by the Laboratory of Reproductive Andrology, West China Second University Hospital of Sichuan University (WCSUH-SCU), China, which is an ISO9001 certified laboratory. The study protocol was approved by the ethics review board of WCSUH-SCU (IRB No. 2020–102).

The EQA program was organized in June of each year and consisted of two semen aliquots, with a total of four samples sent out over two years, numbered 2021A, 2021B, 2022A and 2022B. Each participating laboratory received different aliquots from the same batch of samples and returned their results to the WCSUH- SCU.

The results of the participants were analyzed by WCSUH-SCU. Feedback and improvement suggestions were provided to the participating laboratories through annual participants’ meetings.

### Sample preparation and distribution

The Laboratory of Reproductive Andrology prepared all samples for this EQA program. The samples were obtained from different patients after an abstinence period of 2 to 7 days. The ejaculates that remained after semen analysis were mixed to provide two lots: one with a high SDF value (≥ 30%) and one with a low SDF value (< 30%). The pooled semen samples were evenly distributed in cryotubes, each containing 500 µl of semen, and then directly immersed in liquid nitrogen for preservation. Samples were transported to each laboratory in dry ice. The distance to the farthest laboratory was approximately 3,000 km, and samples could be transported to the laboratory within three days. Participants were asked to test EQA samples according to the laboratory’s routine patients sample procedure.

The homogeneity and the stability of the samples were assessed according to ISO13528 (Statistical methods for use in proficiency testing by interlaboratory comparisons, 2015, Geneva, Switzerland) norms [17]. Before sample shipment, 20 samples were randomly removed from liquid nitrogen, and 10 samples were thawed to detect SDF by the SCSA method. Each sample was tested twice to assess the homogeneity of the samples.

**Fig. 1:**
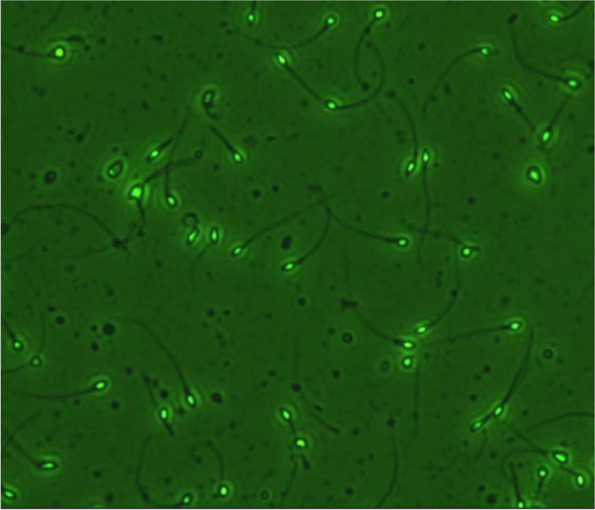
Distribution of spermatozoa under a microscope after thawing. Phase-contrast microscopy was performed with a green filter at 20 × magnification. Spermatozoa were unstained

The remaining 10 samples were placed at -80℃, and the SDF values of these 10 samples were tested after 5 days to assess the stability of the samples at ‒80℃. After thawing, the samples were thoroughly mixed and examined microscopically, which showed a uniform distribution of spermatozoa without agglutination (Fig. 1).

### Data collection and processing

Participating laboratories were required to submit the results within two weeks of receiving the samples. Obtained test results were recorded as the percentage of SDF, and additional information was also provided about the test methods used and the brands of instruments and reagents. The EQA results were reported as the target value, standard deviation (SD) and bias. The target value refers to the mean of all participant results after excluding outliers. Bias was calculated according to the following formula: Bias = (participant result ‒ target value)/target value × 100%.

### Statistical analyses

The homogeneity of samples was evaluated by one-way analysis of variance (ANOVA). The stability of the samples was assessed using an independent sample t-test. The results of the EQA scheme were presented as the mean and standard deviation per sample. The CV for each sample was calculated. In addition, CV values, medians and upper and lower quartiles (Q1-Q3) of SCSA and SCD were calculated. The Kruskal–Wallis test was then performed to compare the results of SCSA and SCD. The range of results for the four samples was summarized in boxplots. For statistical analysis, we used IBM SPSS Statistics version 25.0 (IBM Corporation, Armonk, NY, USA). A p value less than 0.05 was considered statistically significant.

## Results

### Basic information about participants

Thirteen cities in 6 provinces (Gansu, Shanxi, Sichuan, Chongqing, Xinjiang, Yunnan) participated in the EQA in 2022. The types of laboratories participating in EQA are mainly andrology laboratories and some clinical laboratories. The participating hospitals are mainly tertiary hospitals, with a small number of primary and secondary hospitals. All participants in the EQA used SCSA and SCD. The use of SCSA method increased from 50% in 2021 to 60.7% in 2022 (Table 1).

**Table 1:** Basic information about laboratories participating in the external quality assessment

Basic information	2021	2022
Number of participants	10	28
Geographical distribution		
Provinces	2	6
Cities	5	13
Type of laboratories		
Andrology laboratory	8/10 (80.0%)	25/28 (89.3%)
Clinical laboratory	2/10 (20.0%)	3/28 (10.7%)
Hospital grade		
Tertiary hospital	8/10 (80.0%)	24/28 (85.6%)
Secondary hospital	1/10 (10.0%)	2/28 (7.2%)
Primary hospital	1/10 (10.0%)	2/28 (7.2%)
Detection Method		
SCSA	5/10 (50.0%)	17/28 (60.7%)
SCD	5/10 (50.0%)	11/28 (39.3%)

*SCSA* Sperm chromatin structure assay, *SCD* Sperm chromatin dispersion

### The homogeneity and stability of samples

For the homogeneity of the samples, the p values of 2021A, 2021B, 2022A and 2022B were 0.102, 0.731, 0.492 and 0.537, respectively (Supplementary Table 1). For the stability of samples, the *p* values of 2021A, 2021B, 2022A and 2022B were 0.757, 0.060, 0.332 and 0.556, respectively, indicating that the results were stable when the samples were transported at ‒80℃ for 5 days (Supplementary Table 2).

There was no significant difference in SDF between directly frozen semen samples and fresh semen samples (*p* = 0.4, r = 0.9714) (Supplementary Figs. 1 & 2).

### The results of SDF

The results of the four samples are quite different. In 2021, 10 participating laboratories returned the results of 2021A and 2021B, and the highest values were 13.7 and 4.2 times of the lowest values, respectively. In 2022, the highest values of 2022A and 2022B of 28 participating laboratories were 8.0 and 4.0 times of the lowest values, respectively. The range of the high SDF value samples (2021B and 2022A) was larger than that of the low SDF value samples (2021A and 2022B) (Table 2).

**Table 2:** The mean, SD, range and CV of SDF results of four samples

Sample no	Mean (%)	SD	Range (%)	CV (%)
2021A	13.1	6.1	1.6‒21.9	46.6
2021B	46.8	14.1	14.7‒60.4	30.1
2022A	39.7	10.6	6.5‒51.7	26.7
2022B	23.1	7.0	9.4‒37.8	30.3

*SD* Standard deviation, *CV* Coefficient of variation, *SDF* Sperm DNA fragmentation

The CVs were very high in samples with low SDF values (2021A and 2022B). The CVs of the samples with high SDF values (2021B and 2022A) were lower than those of the samples with low SDF values (Table 2).

### Comparison of the SCSA and SCD methods

**Fig. 2:**
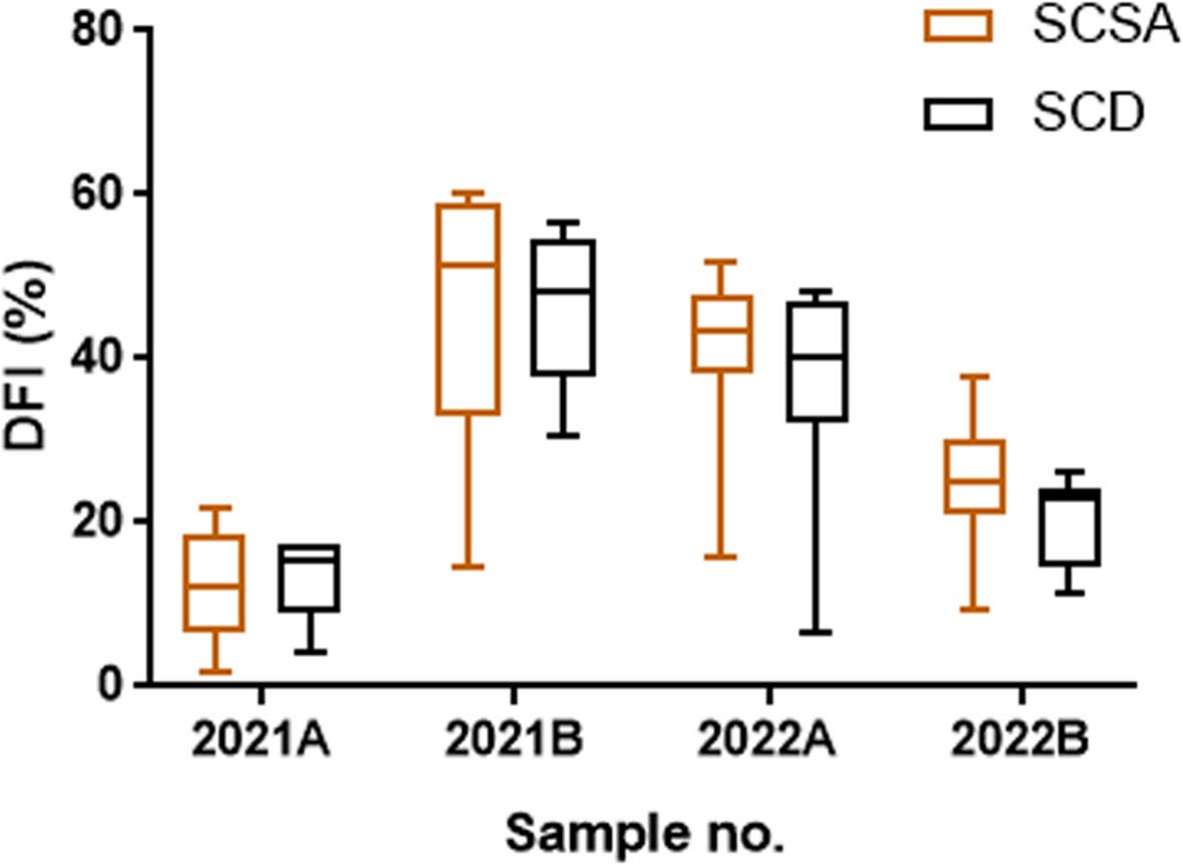
Results of SCSA and SCD for four samples (2021A, 2021B, 2022A and 2022B). The 25th and 75th percentiles are represented by boxes, with the median value, while the 10th and 90th percentiles are represented by whiskers. DFI, DNA fragmentation index; SCSA, sperm chromatin structure assay; SCD, sperm chromatin dispersion

**Fig. 3:**
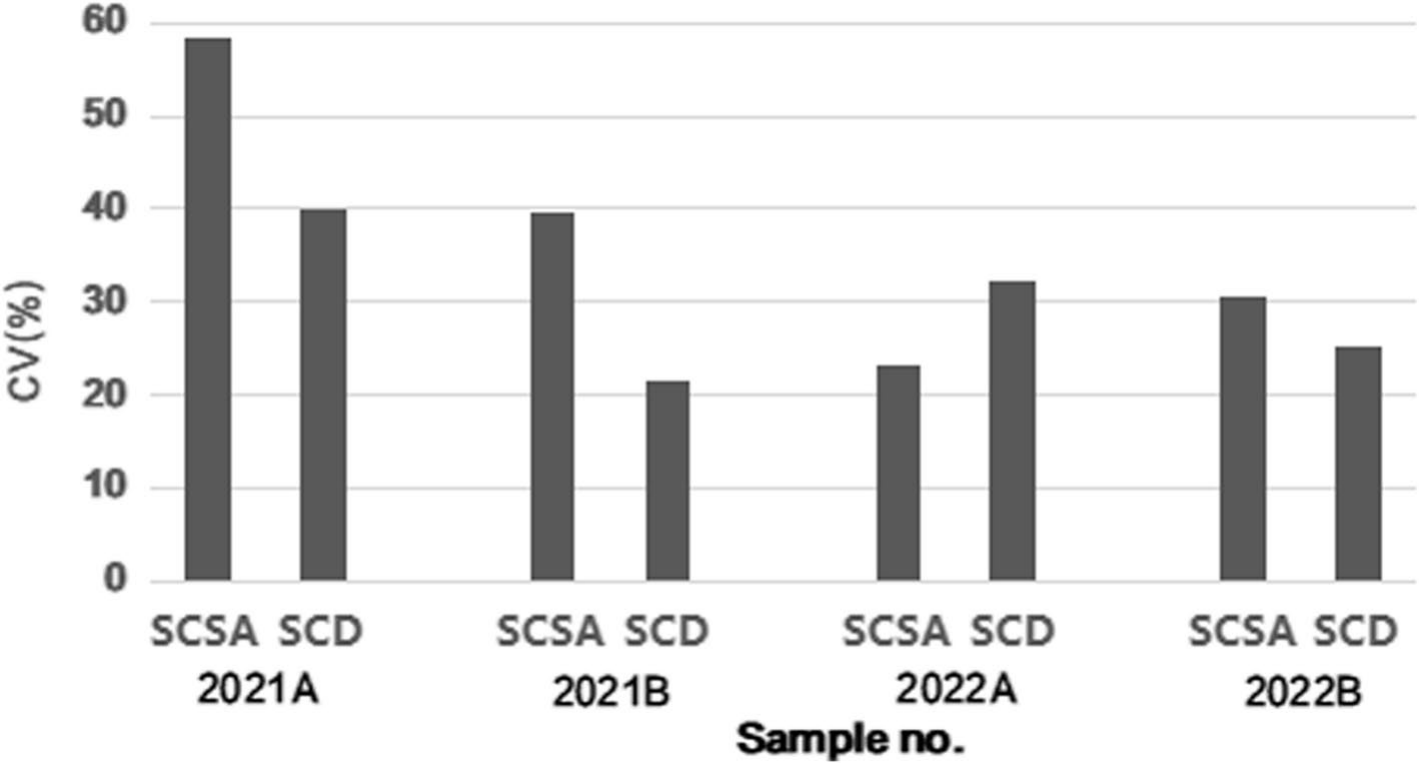
The CVs of SCSA and SCD in four samples (2021A, 2021B, 2022A and 2022B). CV, coefficient of variation; SCSA, sperm chromatin structure assay; SCD, sperm chromatin dispersion

The median results of the four samples tested by the SCSA were 12.0% (Q1‒Q3:6.7‒18.5%), 51.4% (Q1‒Q3:33.0‒59.2%), 43.6% (Q1‒Q3:38.1‒47.7%) and 25.0% (Q1‒Q3:21.1‒30.0%). The median results of the four samples detected by SCD were 15.5% (Q1‒Q3:9.1‒17.5%), 48.0% (Q1‒Q3:37.7‒54.6%), 40.0% (Q1‒Q3:32.3‒47.0%) and 23.0% (Q1‒Q3:14.8‒26.0%), respectively. Except for 2021A, the median results of SCSA were slightly higher than SCD for the remaining three samples (Fig. 2). Furthermore, there was no significant difference between the results of SCSA and SCD in the 4 samples (p = 0.463, 0.465, 0.312, 0.145). The CVs were 58.4%, 39.5%, 23.3% and 30.5% for SCSA and 39.9%, 21.5%, 32.4% and 25.1% for SCD, respectively. Apart from 2022A, the CVs of SCSA were higher than those of SCD (Fig. 3).

### The results of 10 participants in 2021 and 2022

The 10 laboratories that participated in the EQA in 2021 all participated in the EQA in 2022.

For these 10 participants, CVs decreased from 46.6% to 32.5% in the low DFI value samples, and from 30.1% to 22.7% in the high DFI value samples.

**Fig. 4:**
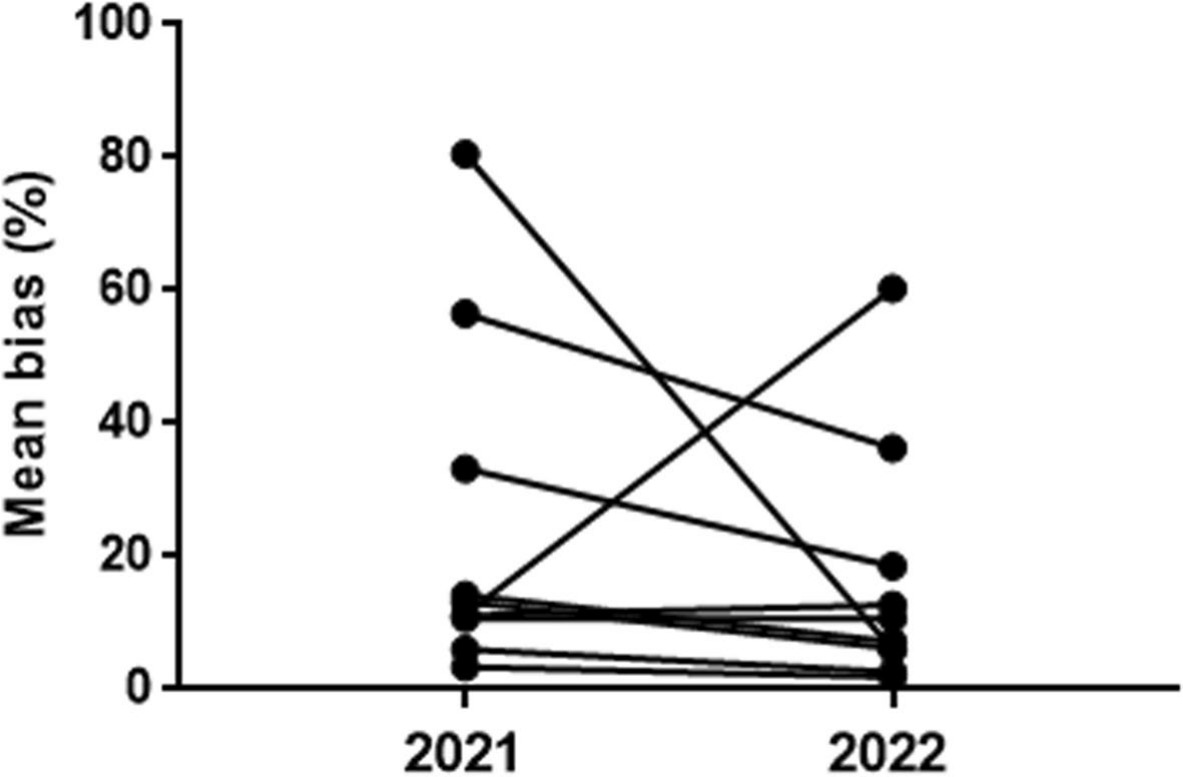
Comparison of the mean bias of 10 laboratories in 2021 and 2022. The 10 points on the left are the mean bias of two samples of 10 participants in 2021. The 10 points on the right are the mean bias of the two samples in 2022 for those 10 participants

The mean bias of two samples from each of the 10 participants in 2021 was compared with the mean bias in 2022; it decreased for 8 participants, with the most significant decreased from 80.5% to 6.0%. There was a slight but insignificant increase in the mean bias in one participant, which increased from 11.3% to 60.3% (Fig. 4). The mean bias of the 10 participants in 2021 was 24%, while it decreased to 16% in 2022.

### Case analysis of participants with abnormal results

#### Case A: the flow cytometer voltage was set too low

##### Case description

In 2021, the target values of the two samples were 13% and 55%, and one of the participants reported 2% and 15%, respectively.

##### Case analysis and solution

**Fig. 5:**
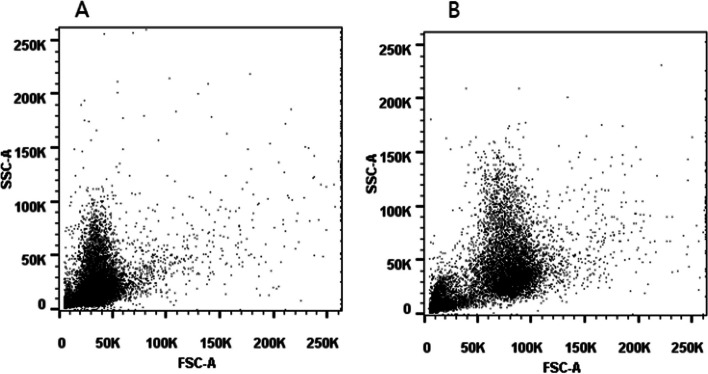
**A** Dot plot with lower voltage settings for flow cytometry. The voltage was low and the boundary of the sub-populations was unclear. **B** Dot plot with normal voltage settings for flow cytometry. The voltage was normal and the boundary of the sub-populations was clear. FSC: forward scatter; SSC: side scatter

The results of two samples were relatively low at the same time, which was considered a systematic error. The causes could be technician operating problems, reagent problems or instrument problems. After analyzing the cause together with the participant's technician, it was found that the boundary between the sperm population and the other population in the FSC/SSC dot plot was not clear, so the reason for the lower results was thought to be the low voltage setting of the FSC channel of the flow cytometer. If the voltage is set too low, the boundary of the sub-populations will be unclear (Fig. 5A), resulting in low detection results. In contrast, the boundary between sub-populations with normal voltage settings should be clear (Fig. 5B). Therefore, the participant's technician worked with the instrument's engineer to adjust the FSC channel voltage, and in 2022, the participant tested 2 specimens with DFI target values of 43% and 22%, and the results were 40% and 21%. Therefore, it is suggested that when using flow cytometry to detect sperm DFI based on SCSA method, first and foremost, the laboratory should set and verify the voltage, so that the different characteristic particle populations can be distinguished well.

#### Case B: The amount of sample added was excessive

##### Case description

In 2022, the target values of the two samples were 43% and 22%, and one of the participants reported 19.6% and 6%, respectively.

##### Case analysis and solution

**Fig. 6:**
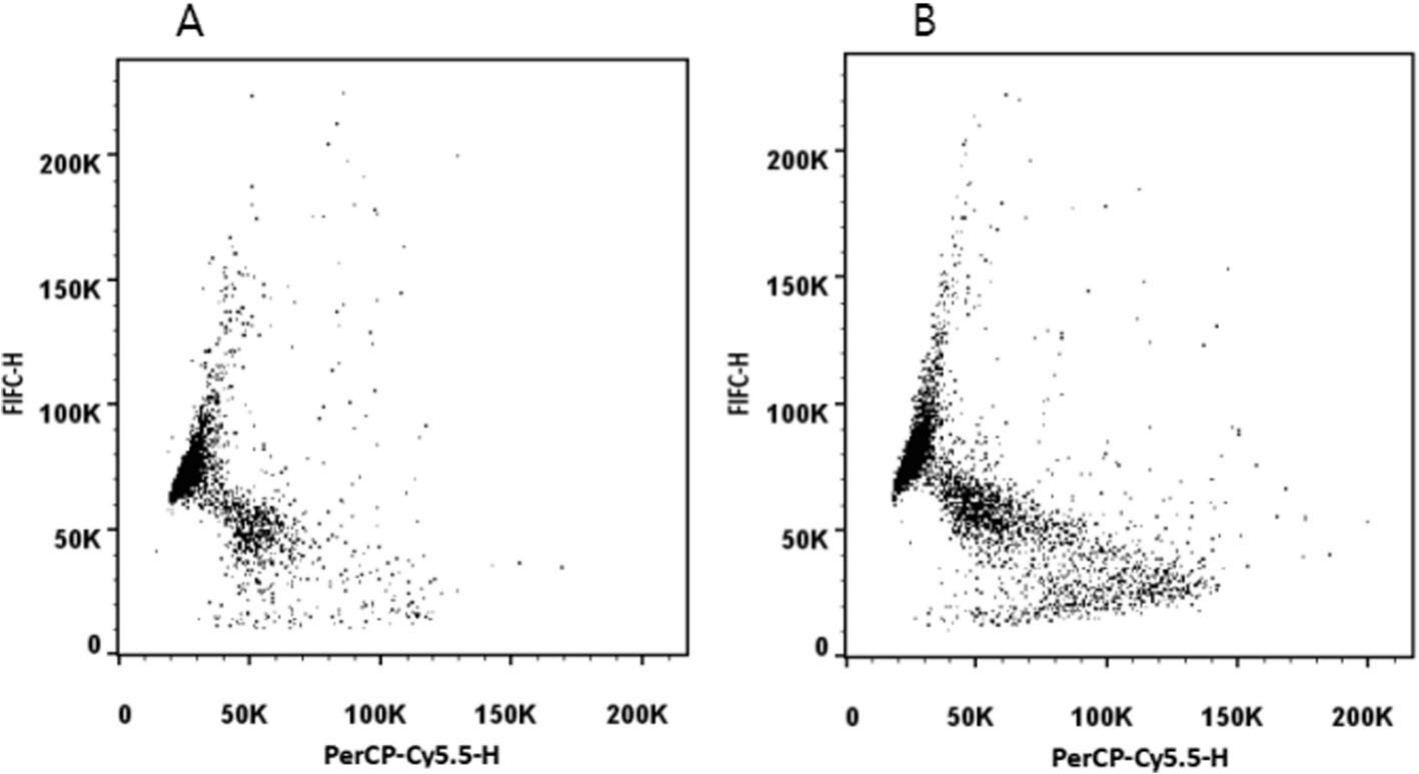
**A** Dot plot with a sample size of 25 µl for flow cytometry. When 25 µl of the semen sample was added, the denaturation effect of the low-pH (1.2) detergent solution on sperm chromatin was weakened, and the result of sperm DNA fragmentation was low. **B** Dot plot with a sample size of 5 μl for flow cytometry. When 5 μl of the semen sample was added, the acid denaturation effect of the acid treatment solution was good and the result of sperm DNA fragmentation was accurate. PerCP-Cy5.5: red AO fluorescence of sperm with broken DNA; FIFC: green AO fluorescence of DNA stainability

The results of both samples were simultaneously low and were considered to be a systematic error. When checking the technician's testing process, it was found that the amount of semen sample added by the technician was 25 μl (Fig. 6A). If too much semen sample is added, the denaturation effect of the low-pH (1.2) detergent solution on sperm chromatin will be weakened. Consequently, the technician adjusted the added sample volume to 5 μl and then tested the sample again, and the result of 2202A increased to 41.5% (Fig. 6B). Therefore, it is suggested that when SDF is detected by flow cytometry, on the one hand, sperm should first be diluted to 1–2 million per milliliter according to the WHO manual, and on the other hand, the amount of semen added should not be too high.

#### Case C: The results were obtained through a fixed gate of software

##### Case description

In 2022, the target values of the two samples were 43% and 22%, and one of the participants reported 24% and 9%, respectively.

##### Case analysis and solution

**Fig. 7:**
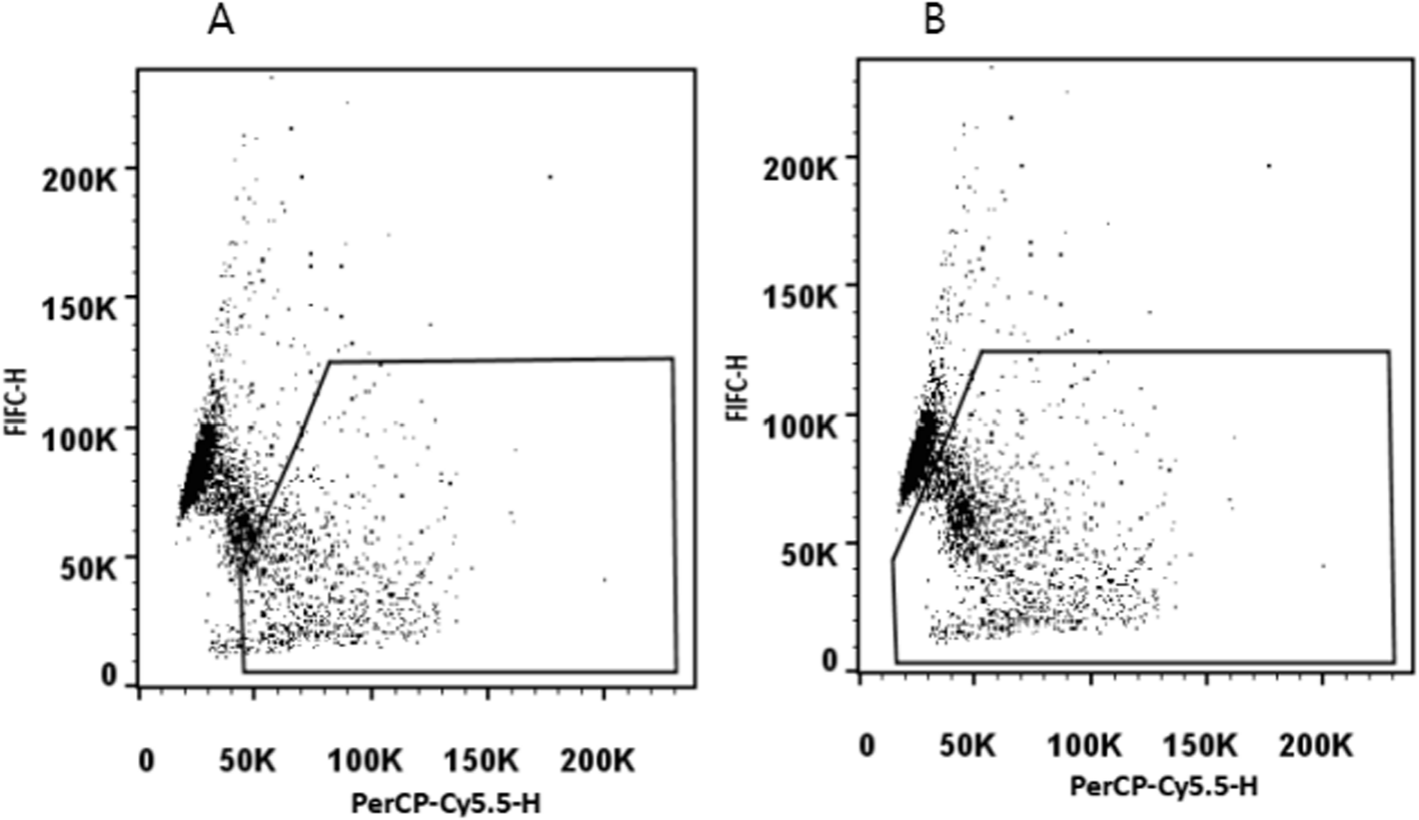
**A** Dot plot with fixed gating analysis for flow cytometry. The fixed gating of the flow cytometry analysis software was biased to the right, and the result of sperm DNA fragmentation was reduced. **B** Dot plot with manual gating analysis for flow cytometry. Sperm with abnormal DNA fragmentation can be well included by manual gating. PerCP-Cy5.5: red AO fluorescence of sperm with broken DNA; FIFC: green AO fluorescence of DNA stainability

The results of the two samples declined at the same time. A search for reasons revealed that the participant used the fixed gate of the flow cytometry analysis software directly to obtain the results (Fig. 7A). Nevertheless, manual gating analysis of the raw data files of 2202A by the organizers yielded results of 44% (Fig. 7B). Although some flow cytometers contain automatic analysis software, manual verification of gating accuracy by laboratories is still recommended.

#### Case D: The flow rate was set too high

##### Case description

In 2022, the target values of the two samples were 43% and 22%, and one of the participants reported 24% and 15%, respectively.

##### Case analysis and solution

**Fig. 8:**
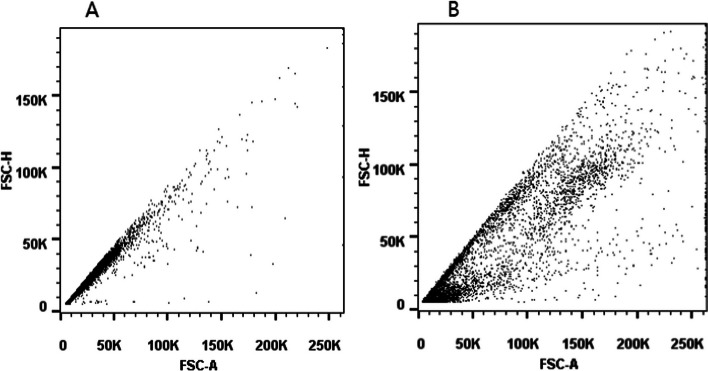
**A** Dot plot with low speed for flow cytometry. At low speed, a single cell passes through the sample tube, and cells appear on a diagonal. **B** Dot plot with high speed for flow cytometry. At high speed, multiple cells pass through the sample tube at the same time, and the cells are clustered together in an off-diagonal distribution. FSC: forward scatter; SSC: side scatter

The results are significantly lower for both samples, which is considered to be a systematic error. After investigation it was found that the flow rate on the flow cytometer was set to high speed when testing the samples, which may cause two or more spermatozoa to pass through the flow chamber of the flow cytometer at the same time, affecting the test results. The flow rate should be set to a low speed (no more than 300 events/s) for SDF detection on the flow cytometer. When a single cell passes through, the FSC-H correlates with the FSC-A and cells appear on a diagonal in the FSC-H/FSC-A dot plot (Fig. 8A). However, when cells clump together, they exhibit an off-diagonal distribution (Fig. 8B).

## Discussion

In the field of reproductive medicine, the clinical significance of SDF is a trending topic. The negative impact of SDF on male fertility potential may prompt more clinicians to utilize this indicator [18]. Therefore, it is critical to perform both EQA and IQC programs to ensure methodological standardization and accuracy of SDF testing, thus providing reliable information for patient diagnosis and treatment. While there have been extensive studies on the standardization and quality control of routine semen analysis, there have been limited studies focused specifically on the standardization and QC of SDF assays [19]. EQA for routine semen analysis was explored in Europe as early as 1990 [20]. However, there are few studies on the standardization and QC of SDF assay [21, 22]. EQA of SDF program has not been reported yet. The present study is the first report to establish such an EQA program.

The sample preparation procedure for EQA in this study was not complicated and the samples used were homogeneous and showed no agglutination. However, it was observed that the cost of transporting samples using dry ice was relatively high. Therefore, further research is needed to identify alternative methods of transportation that can be done at room temperature without compromising the SDF results.

The large inter-laboratory variability in SDF results is a concern as it can have a significant impact on patient diagnosis and treatment. It was found that the same sample could be judged as normal by one laboratory and abnormal by another. To address this issue and achieve better agreement among different laboratories, it is necessary to further standardize the methods for SDF testing and implement strict IQC and EQA programs.

The organizer of the EQA program holds an annual concluding meeting to provide feedback and analysis on the problems encountered by the participants throughout the year. This meeting also includes training on standardized operating procedures and IQC for SDF testing. Additionally, the organizer reaches out to participants whose results significantly differ from the target value to jointly analyze the possible causes and find ways to obtain accurate results.

The study showed that the mean bias of most participants decreased, indicating that the analysis and treatment of EQA problems, along with participant training, have a positive effect on reducing the variability of SDF results. However, it was observed that one participant reported setting high flow rate during sample testing, which significantly increased the mean bias. This emphasizes the importance of proper training and adherence to standardized operating procedures to ensure accurate and reliable SDF results.

The four cases mentioned in the results were specific examples that were used to illustrate the process of identifying and addressing the factors that have an impact on the overall results obtained. These factors, which were discovered to be responsible for the consistently low results, were exclusively found in the SCSA assay. It is worth noting that in China, SDF evaluation is mainly based on SCD and SCSA. Among these, SCSA is the preferred choice for SDF testing in China due to its rapid analysis, simple operation, and high level of accuracy. Traditionally, SCSA results have been considered to be more precise and reliable compared to SCD results. However, the findings of this study indicate that SCSA is more susceptible to systematic errors than SCD. The CV values of SCSA, except for 2022A, were higher than those of SCD. Factors such as sample size and flow cytometry parameter settings (voltage, flow rate and gate, etc.) could contribute to occurrence of systematic errors in the SCSA method. Consequently, it is crucial for laboratories opting for SCSA as a method for detecting SDF to ensure the appropriateness of their flow cytometry parameter settings. Moreover, other factors that can potentially influence SDF detection have been reported in the literature, indicating that the duration of acid denaturation is a critical factor and should not exceed one minute at most, and that the samples used for sperm DFI detection should be fresh and not exceed one day in case of refrigeration [23].

In this study, it was observed that the median results obtained from SCSA were slightly higher than those from SCD in three out of the four samples, which aligns with the findings from some previous studies [24]. The previous study showed that the difference in sperm DFI values between SCSA (26.98 ± 1.28%) and SCD (27.88 ± 1.278%) was not statistically significant (p > 0.05). Nevertheless, there are other studies that suggest SCD and SCSA provide distinct information, leading to conflicting results between the two methods [15, 16]. Despite the methodological disparities between SCD and SCSA, several researchers have indicated that both methods use similar threshold levels to determine the extent of sperm DNA damage [25]. In fact, a recent meta-analysis conducted by Santi concluded that an SDF cut-off value of 20% exhibits good predictive power in distinguishing fertile males from those experiencing infertility [26].

## Conclusion

This is the first study to evaluate the EQA program on SDF, and it involved multiple laboratories to ensure comprehensive results. The study successfully demonstrated the feasibility of the EQA program. One of the key aspects of this study was the distribution of native samples to the participating laboratories, which allowed the analyses to be carried out under routine conditions, closely mimicking the performance of the tests in real-life practice. The SDF results, obtained from various laboratories, showed significant discrepancies, highlighting the importance of training for enhancing the test performance. To enhance the reliability and consistency of the results, it is recommended that laboratories implement IQC measures. Additionally, to ensure the accuracy and comparability of the SDF test results, it is essential for all laboratories to participate in the EQA program. This ensures consistent and reliable test results across different laboratories, instilling confidence in the accuracy of the results.

### Supplementary Information

**Additional file 1:** **Supplementary Fig. 1.** Comparison of DFI between fresh semen and frozen semen. Frozen semen refers to semen stored directly in liquid nitrogen. There was no significant difference with a p value of 0.4 by paired t-test .The 25th and 75th percentiles are represented by boxes, with the median value, while the 10th and 90th percentiles arerepresented by whiskers. DFI, DNA fragmentation index. **Supplementary Fig. 2.** Correlation of DFI results between fresh and frozen semen. Frozen semen refers to semen stored directly in liquid nitrogen. Linear correlation was used for statistics (r =0.9714). DFI, DNA fragmentation index. **Supplementary Table 1.** The homogeneity of samples. **Supplementary Table 2.** The stability of samples stored at -80°C for the first and fifth days.

## Data Availability

The datasets generated and analyzed during the current study are not publicly available because local institutional patient data are considered confidential but are available from the corresponding author upon reasonable request.

## Abbreviations

EQA: External quality assessment
SDF: Sperm DNA fragmentation
CV: Coefficient of variation
SCSA: Sperm chromatin structure assay
SCD: Sperm chromatin dispersion
IQC: Internal quality control
SD: Standard deviation
DFI: DNA fragmentation index
