# Supplementary material for: External quality assessment scheme for sperm DNA fragmentation: a pilot study in China
Source: Basic Clin Androl. 2023 Nov 28;33:36. doi: 10.1186/s12610-023-00211-0 (PMC10683090; doi:10.1186/s12610-023-00211-0)
Supplement: Supplementary file 1 — Additional file 1: Supplementary Fig. 1. Comparison of DFI between fresh semen and frozen semen. Frozen semen refers to semen stored directly in liquid nitrogen. There was no significant difference with a p value of 0.4 by paired t-test .The 25th and 75th percentiles are represented by boxes, with the median value, while the 10th and 90th percentiles arerepresented by whiskers. DFI, DNA fragmentation index. Supplementary Fig. 2. Correlation of DFI results between fresh and frozen semen. Frozen semen refers to semen stored directly in liquid nitrogen. Linear correlation was used for statistics (r =0.9714). DFI, DNA fragmentation index. Supplementary Table 1. The homogeneity of samples. Supplementary Table 2. The stability of samples stored at -80°C for the first and fifth days. [file 12610_2023_211_MOESM1_ESM.doc]

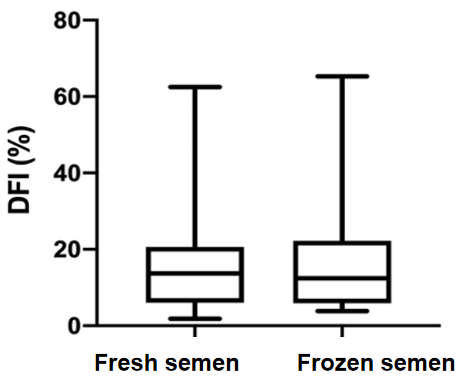


Supplementary Fig. 1 Comparison of DFI between fresh semen and frozen semen. Frozen semen refers to semen stored directly in liquid nitrogen. There was no significant difference with a p value of 0.4 by paired t-test .The 25th and 75th percentiles are represented by boxes, with the median value, while the 10th and 90th percentiles arerepresented by whiskers. DFI, DNA fragmentation index.


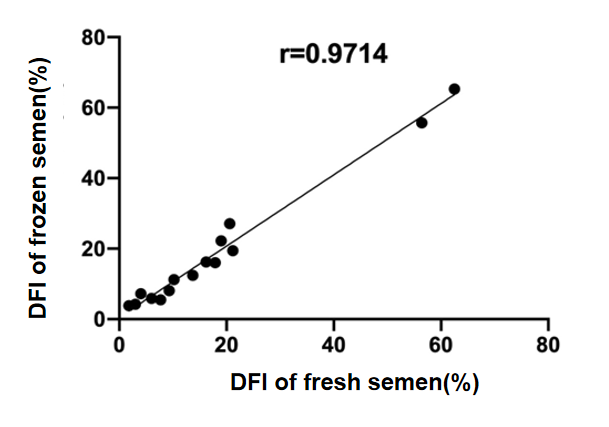


Supplementary Fig. 2 Correlation of DFI results between fresh and frozen semen. Frozen semen refers to semen stored directly in liquid nitrogen. Linear correlation was used for statistics (r =0.9714). DFI, DNA fragmentation index.

Supplementary Table 1 The homogeneity of samples.

| Sample no. | 2021A DFI(%) | | 2021B DFI(%) | | | 2022A DFI(%) | | | 2022B DFI(%) | | |
| --- | --- | --- | --- | --- | --- | --- | --- | --- | --- | --- | --- |
|  | First | Second | | First | Second | | First | Second | | First | Second |
| 1 | 11 | 15 | | 45 | 52 | | 45 | 40 | | 24 | 25 |
| 2 | 10 | 11 | | 44 | 47 | | 43 | 36 | | 24 | 25 |
| 3 | 10 | 11 | | 50 | 45 | | 44 | 42 | | 25 | 25 |
| 4 | 10 | 13 | | 47 | 44 | | 45 | 42 | | 25 | 22 |
| 5 | 13 | 12 | | 46 | 47 | | 44 | 44 | | 22 | 21 |
| 6 | 14 | 15 | | 46 | 48 | | 43 | 38 | | 24 | 24 |
| 7 | 11 | 11 | | 49 | 45 | | 43 | 37 | | 26 | 22 |
| 8 | 13 | 14 | | 50 | 42 | | 44 | 40 | | 23 | 23 |
| 9 | 14 | 16 | | 57 | 57 | | 37 | 39 | | 24 | 23 |
| 10 | 12 | 14 | | 56 | 56 | | 37 | 41 | | 22 | 25 |

Ten samples of 2021A, 2021B, 2022A and 2022B were randomly selected to detect DFI. Each sample was tested twice using one-way ANOVA to assess the homogeneity. The P values of 2021A, 2021B, 2022A and 2022B were 0.102, 0.731, 0.537 and 0.492, respectively. DFI, DNA fragmentation index.

Supplementary Table 2 The stability of samples stored at -80°C for the first and fifth days.

| Sample no. | DFI（%） on the first day | DFI （%）on the fifth day | p-Value |
| --- | --- | --- | --- |
| 2021A | 12.5±1.6 | 12.8±1.5 | 0.757 |
| 2021B | 48.7±4.3 | 46.9±1.8 | 0.060 |
| 2022A | 42.5±2.8 | 41.2±2.0 | 0.556 |
| 2022B | 23.9±1.3 | 23.7±1.0 | 0.332 |

Values are presented as mean±standard deviation. There was no significant difference between the results of the samples stored at -80°C for the first and fifth days using a paired t-test. The p values of 2021A, 2021B, 2022A and 2022B were 0.757, 0.060, 0.332 and 0.556, respectively. DFI, DNA fragmentation index.
